# Myosin light chain phosphorylation exhibits a gradient across the wall of cerebellar arteries under sustained ex vivo vascular tone

**DOI:** 10.1038/s41598-023-28092-3

**Published:** 2023-01-17

**Authors:** Zhe Sun, Zhaohui Li, Mackenna Rodgers, Liping Zhang, Michael A. Hill

**Affiliations:** 1 Dalton Cardiovascular Research Center, University of Missouri, Columbia, MO 65211 USA; 2 Department of Medical Pharmacology and Physiology, School of Medicine, University of Missouri, Columbia, MO USA; 3 Department of Radiology, School of Medicine, University of Missouri, Columbia, MO USA

**Keywords:** Blood flow, Cardiovascular biology

## Abstract

Small blood vessel diseases are often associated with impaired regulation of vascular tone. The current understanding of resistance arteries often focuses on how a level of vascular tone is achieved in the acute phase, while less emphasis is placed on mechanisms that maintain vascular tone. In this study, cannulated rat superior cerebellar arteries (SCA) developed spontaneous myogenic tone and showed a marked and sustained constriction in the presence of diluted serum (10%), a stimulus relevant to cerebrovascular disease. Both phosphorylated myosin light chain (MLC-p) and smooth muscle alpha actin (SM-α-actin) aligned with phalloidin-stained actin filaments in the vessel wall, while exhibiting a ‘high to low’ gradient across the layers of vascular smooth muscle cells (VSMC), peaking in the outer layer. The MLC-p distribution profile shifted towards the adventitia in serum treated vessels, while removal of the serum reversed it. Furthermore, a positive correlation between the MLC-p signal and vessel wall tension was also evident. The gradients of phosphorylated MLC and SM-α-actin are consistent with a spatial regulation of the myosin-actin apparatus in the vessel wall during the maintenance of vascular tone. Further, the changing profiles of MLC-p and SM-α-actin are consistent with SCA vasoconstriction being accompanied by VSMC cytoskeletal reorganization.

## Introduction

Chronic vascular disorders, such as hypertension, are often associated with pathology and dysfunction of distal arteries^1^, referred to as small vessel disease (SVD). SVD is usually manifest as a narrowing of resistance arteries, ultimately restricting end organ blood flow and promoting ischemia. Importantly, in the aging population, SVD is a major contributor to cerebrovascular pathology, including lacunar stroke and vascular dementia leading to cognitive impairment^2^. Mechanistically, an exaggerated small artery myogenic response (vasoconstriction to increased intraluminal pressure)^3,4^ and endothelial cell dysfunction^5,6^ are two putative causes of excessive small vessel constriction. To date, our understanding of the myogenic response has predominantly focused on how pressure acutely induces vasoconstriction^7,8^, with relatively less emphasis being placed on how a small artery maintains its physiological diameter over the longer timeframe, referred to as myogenic tone. Vascular tone in small arteries is predominantly regulated by Ca^2+^-induced contraction of the actin-myosin myofilament apparatus. In this regard, Ca^2+^ mobilization in small artery vascular smooth muscle has been extensively studied in the past few decades^9–12^. More recently, mechanisms relating to actin cytoskeleton remodeling have been shown to also play an important role in the regulation of vasomotor tone and are likely to be mediated by mechanisms distinct from acute Ca^2+^ mobilization^13–16^. In addition to these processes, re-elongation of vascular smooth muscle cells (VSMCs) in the vessel wall^17^, and cross-linking and remodeling of extracellular matrix components^18^, have also been proposed to play important roles in sustained vasoconstriction or maintaining vascular tone^19^.

In addition to enhanced myogenic tone in chronic conditions such as aging and hypertension, common cerebrovascular abnormalities can arise from ischemic stroke and subarachnoid hemorrhage (SAH), with the latter often inducing severe brain damage with debilitating outcomes^20^. In addition to delayed vasospasm^20,21^, several novel mechanisms have been proposed that likely contribute to the extent of brain damage after SAH, including early brain injury (EBI)^22,23^, intravascular inflammation^24^ and impaired regulation of the microcirculation^25^. Acute spasm of small cerebral vessels has been observed in rodent SAH models in vivo^26–28^. Further, leakage of blood and diffusion of serum through the subarachnoid space in SAH can be mimicked in vitro by the addition of serum to isolated and cannulated cerebral arteries. In our own preliminary studies, the presence of diluted serum was observed to induce significant and sustained vasoconstriction of small cerebral arteries ex vivo, which could potentially contribute to the acute cerebral vasospasm in experimental SAH.

In the current study, immunofluorescence microscopy was used to examine myosin light chain (MLC) phosphorylation and smooth muscle alpha actin (SM-α-actin) in the vessel wall of small cerebral arteries that were fixed at a stable level of myogenic or serum-induced tone. This approach provided a level of spatial resolution not possible in classical western blotting assays and allowed us to test the novel hypothesis that sustained serum-induced contraction of cerebral arteries was associated with contractile protein reorganization. Our results demonstrate that in cannulated rat superior cerebellar arteries (SCA), the distributions of both phosphorylated MLC and SM-α-actin actin filaments follow a transmural gradient that peaks at the outer layer of vascular smooth muscle (VSM) and reaches a minimum towards the inner layer of VSMC. Serum treatment modified the transmural gradient of phosphorylated MLC. These results are consistent with small arteries maintaining a stable level of tone through mechanisms involving MLC and actin organization that differ from, and likely support, mechanisms underlying the acute phase of vasoconstriction.

## Results

### Distribution of MLC phosphorylation and SM-α-actin within the vessel wall

**Figure 1:**
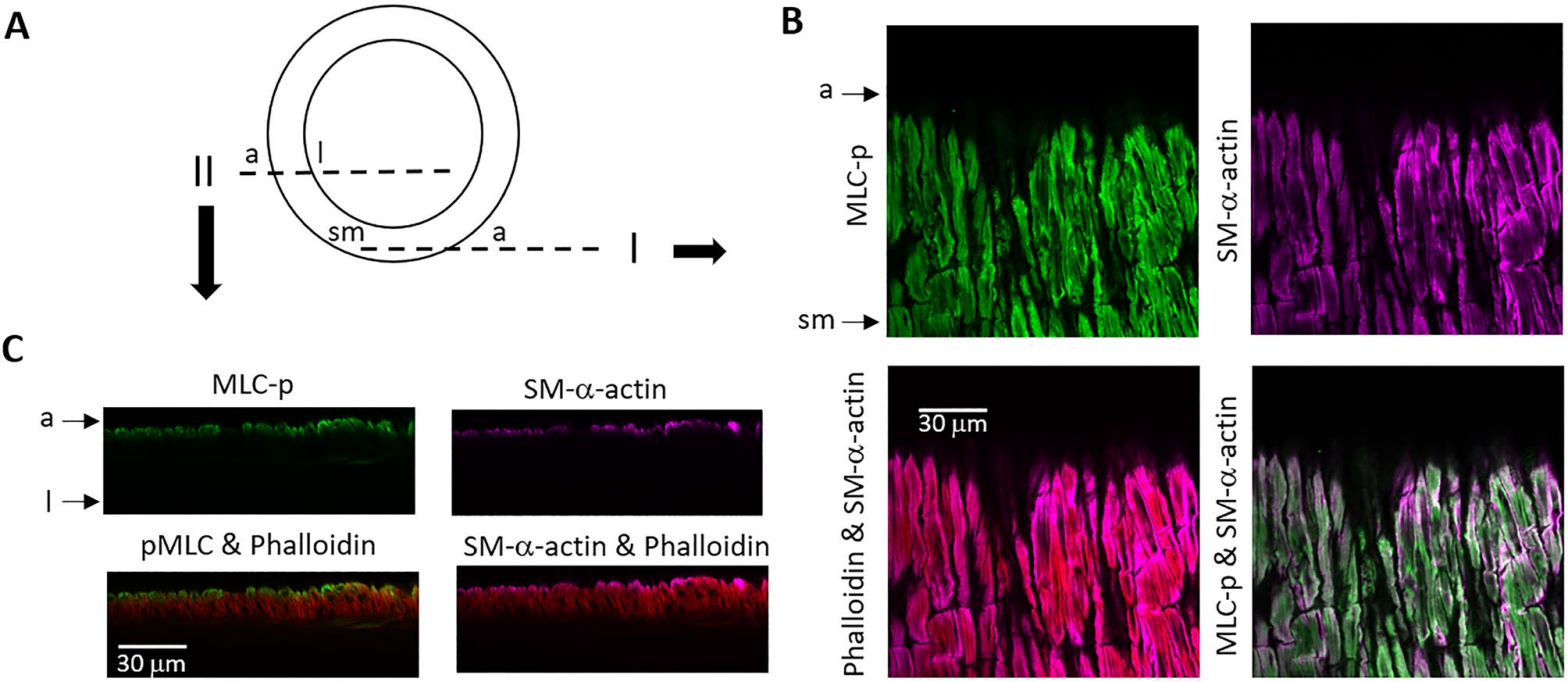
Gradient distribution of MLC-p and SM-α-actin in the vessel wall of SCA. (**A**) Illustration of the longitudinal circumferential view (I) and the longitudinal sectional view (II) of the vessel wall. (**B**) Longitudinal circumferential view showed good correlation among MLC-p, SM-α-actin and phalloidin-labeled actin fiber in SCA that was fixed while maintaining a stable myogenic tone. The vessel was labeled for phosphorylated myosin light chain (MLC-p, green), SM-α-actin (purple) and phalloidin (red). Phalloidin staining was used to identify the smooth muscle layer. Arrows: a: depicts adventitia side of vessel wall; sm: smooth muscle. (**C**) The gradient distribution of MLC-p and SM-α-actin in the vessel wall is evident in the longitudinal sectional view of the SCA, i.e. strong signal in the outer layer, but less intense signal in the inner layer, with phalloidin-labeled actin filaments distributed across the thickness of the vessel wall. a: depicts adventitia side of vessel wall; l: depicts intra-luminal side of vessel wall.

Immunofluorescence microscopy (Fig. 1A) was employed to visualize the distribution of myosin light chain phosphorylation (MLC-p) and SM-α-actin in relation to phalloidin labeled actin filaments in SCAs under a stable level of myogenic tone (Fig. 1B,C). A longitudinal circumferential view of the vessel wall (outer layer view) revealed that both MLC-p and SM-α-actin exhibit fibrillary structures and are well correlated with each other, as well as with the phalloidin labeled actin fibers (Fig. 1B), suggesting that the MLC-p is associated with actin filaments in the vessel wall, as would be expected. Surprisingly, while phalloidin labeled actin fibers can be seen across the thickness of vessel wall, both MLC-p and SM-α-actin were concentrated near the outer edge of the blood vessel wall with only very weak signals being observed in the inner layers of the vessel wall (Fig. 1C). See Fig. 4 for image quantification. Western blotting was used to confirm the specificity of the antibodies used for immunolabeling of MLC-p and SM-α-actin (Supplemental Fig. 1). Immunolabeling performed with rabbit and mouse IgG isotype control antibodies showed negligible fluorescence signals (Supplemental Fig. 1), supporting the specificity of the MLC-p and SM-α-actin immuno-staining in the vessel wall.

### Concentration of MLC-p and SM-α-actin labeling in the outer layer of VSM is not caused by poor antibody penetrance

**Figure 2:**
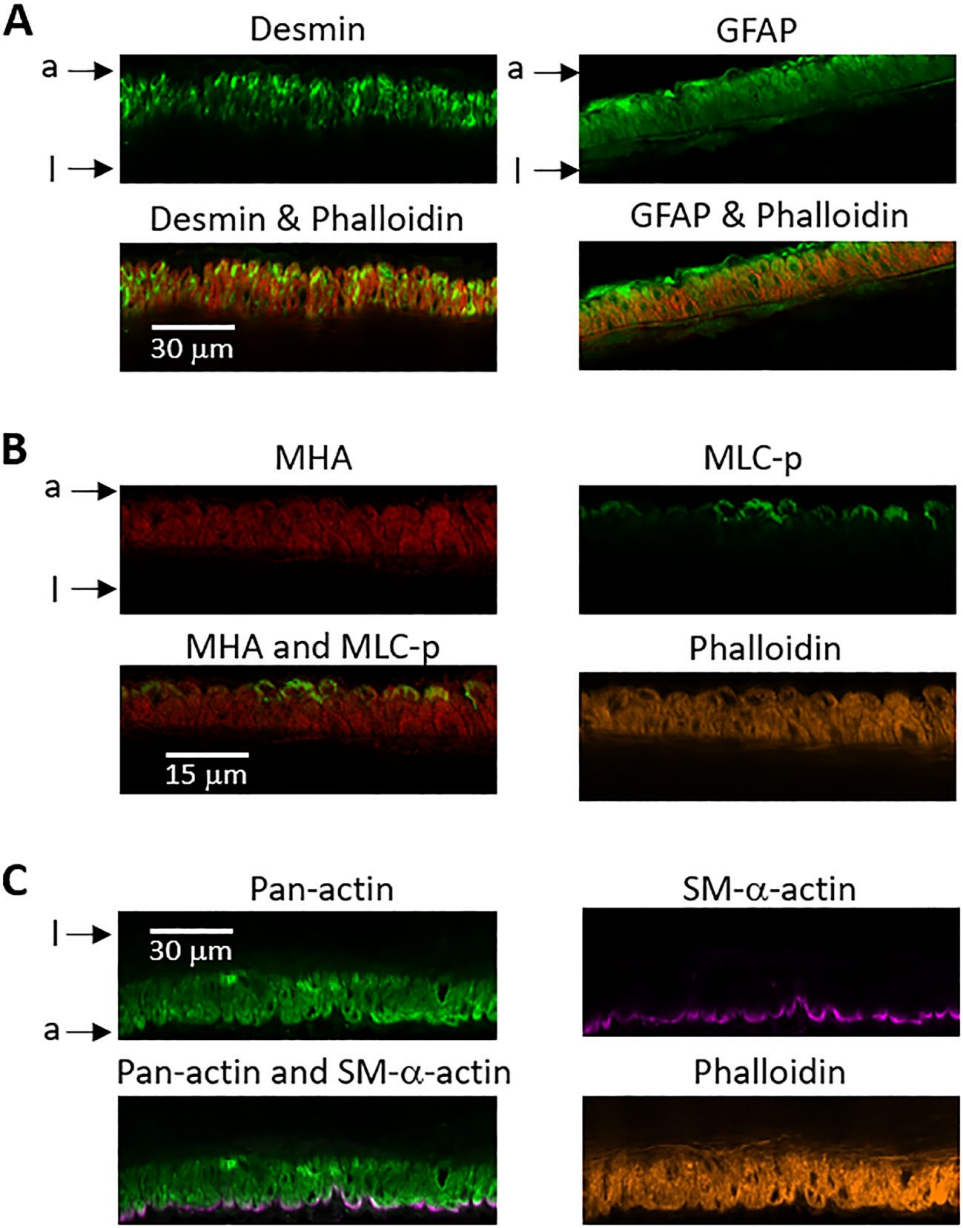
The pattern of MLC-p and SMA-α-actin staining is not due to limited antibody diffusion across the vessel wall. (**A**) Immuno-staining of desmin (green) and GFAP (green) in the vessel wall of rat SCA. Vessel was fixed while maintaining a stable myogenic tone at 70 mmHg. (**B**) Triple staining of myosin heavy chain type A (MHA, red), MLC-p (green) and phalloidin (yellow) in the vessel wall of SCA. (**C**) Triple-staining of pan-actin (green), SM-α-actin (purple) and phalloidin (yellow) in the vessel wall of SCA. a: depicts adventitia side of vessel wall; l: depicts intra-luminal side of vessel wall.

Since both the MLC-p and SM-α-actin labeling were concentrated in the outer VSMC layer, we asked if the labeling pattern could be due to limited diffusion/penetrance of the antibodies into the vessel wall. We therefore compared MLC-p- and SM-α-actin labeling with that of other VSMC proteins. Specifically, the same protocol was used to label desmin and glial fibrillary acidic protein (GFAP) (Fig. 2A and Supplemental Fig. 2A,B) in SCAs, both of which are associated with cytoskeleton function in cerebral VSMC^29,30^. While the distribution of desmin is more discrete and GFAP is more diffuse, labeling for both proteins was apparent across the whole thickness of vessel wall. Dual labeling experiments were also employed to compare the distribution of MLC-p with that of myosin heavy chain A (MHA), and to compare SM-α-actin with that of pan-actin. (Fig. 2B,C and Supplemental Fig. 2C) The concentrated patterns of MLC-p and SM-α-actin labeling were not observed when antibodies against MHA and pan-actin were used, strongly suggesting that the labeling patterns for MLC-p and SM-α-actin were not due to poor antibody penetrance during the staining procedures.

### Serum induced rapid vasoconstriction of rat superior cerebellar artery (SCA)

**Figure 3:**
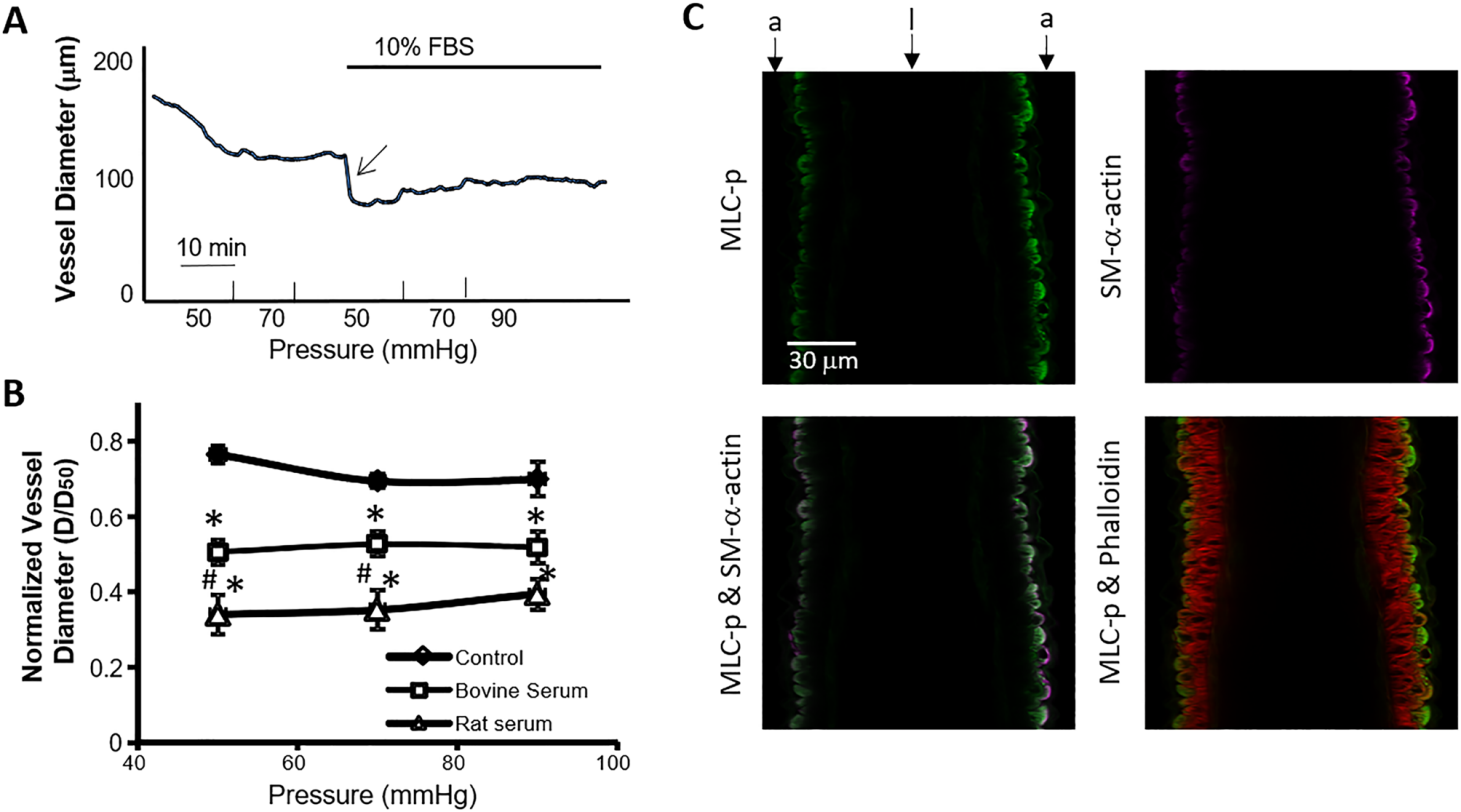
Serum (10%) induced vasoconstriction of SCA. (**A**) A typical recording of SCA diameter at indicated intraluminal pressures before (control) and after exposure to 10% serum. Arrow depicts the robust vessel constriction after serum treatment. Bar shows time scale. (**B**) Effect of exposure to 10% serum on SCA myogenic tone. D: vessel diameter 10 min after pressure elevation; D_50_: passive vessel diameter at 50 mmHg. *p < 0.05 compared to control. Control: n = 6; bovine serum: n = 4; rat serum: n = 6. #*p* < 0.05 compared to Bovine serum. (**C**) Representative longitudinal sectional view of SCA maintaining a steady state vasoconstriction after exposure to 10% rat serum. The gradient distribution of MLC-p (green) and SM-α-actin (purple) in the vessel wall is evident in the wall of serum treated SCA, as compared to the phalloidin staining (red). a: depicts adventitia side of vessel wall; l: depicts intra-luminal side of vessel wall.

In SAH, when blood leaks into the subarachnoid space, where blood cells likely coagulate, the soluble blood-borne factors (serum) could potentially diffuse through the cerebral fluid, affecting both neuronal tissues and blood vessels that are bathed in the cerebral fluid. To mimic this condition ex vivo, we tested the effect of 10% serum on the myogenic tone of rat SCA at 70 mmHg. As shown in Fig. 3A, addition of 10% serum induced significant and sustained vasoconstriction. Interestingly, application of rat serum caused significantly more vasoconstriction than bovine serum (Fig. 3B). Consequently, rat serum was used in the subsequent experiments. Immunofluorescence microscopy was then used, as above, to examine the distributions of MLC-p, SM-α-actin and actin filaments in SCAs constricted by rat serum. As shown in Figs. 3C, the graded distribution of labeling for both MLC-p and SM-α-actin remained apparent.

### Comparison of the effects of serum treatment on SCA MLC-p and actin filaments

**Figure 4:**
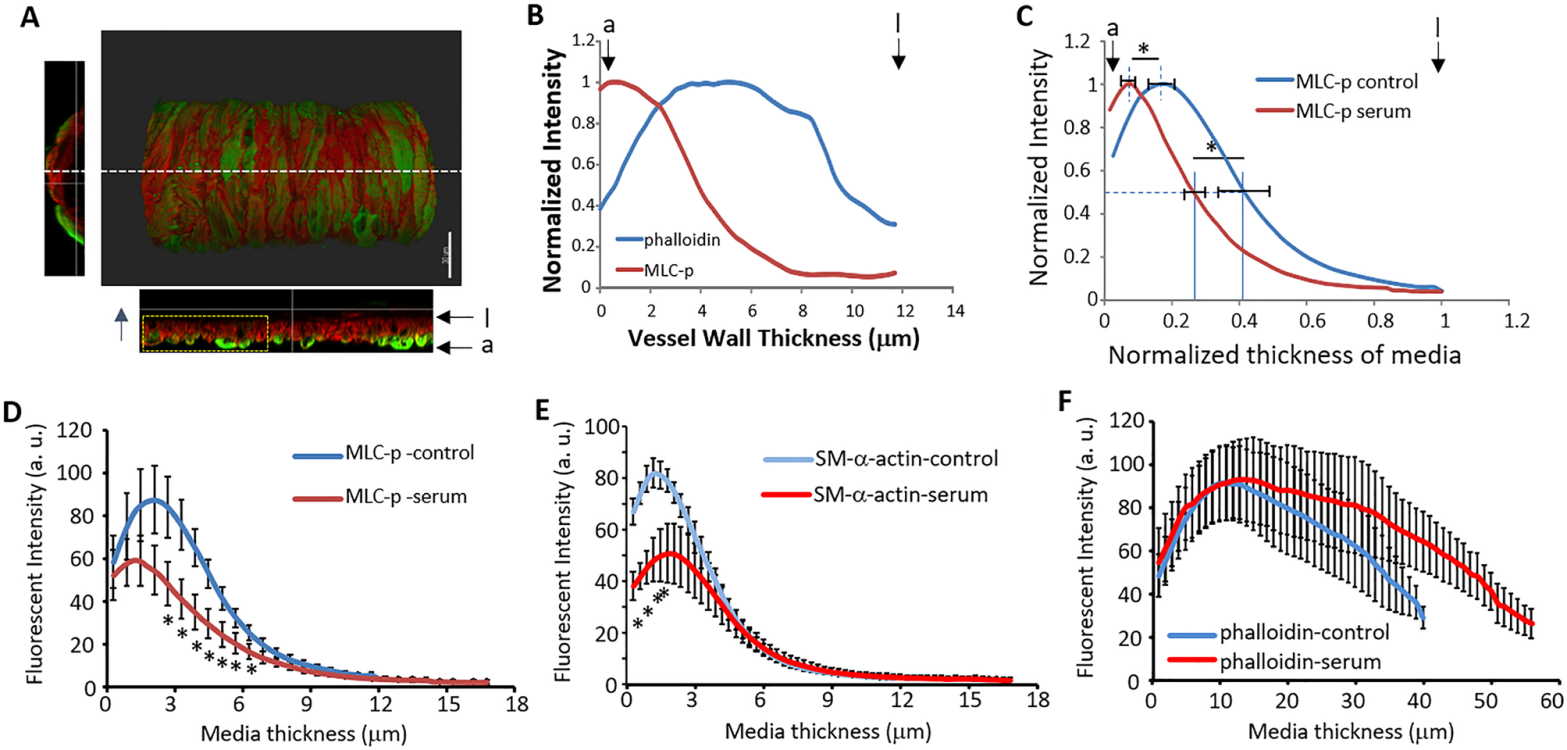
Comparison of the staining patterns for MLC-p, SM-α-actin and phalloidin between control and serum constricted SCA. (**A**) 3D longitudinal view of an immuno-labeled SCA vessel, green-MLC-p, red-phalloidin. White dashed line depicts the plane where a longitudinal orthogonal section was taken, the normal view of the longitudinal section is shown in the bottom image. The section of vessel outlined by the yellow box was used to analyze the intensity profile of MLC-p and phalloidin-labeling across the vessel wall in the direction depicted by the blue arrow , Scale bar = 30 μm. (**B**) The graph depicts the normalized intensity profile of MLC-p and phalloidin of the vessel orthogonal section shown in (**A**), a: depicts adventitia side of vessel wall; l: depicts intra-luminal side of vessel wall. (**C**) At 70 mmHg, the vasoconstriction induced by 10% rat serum is accompanied by a shift of the MLC-p profile to the left (e.g. towards the adventitial edge) of the smooth muscle layer. Dashed line indicates the level of half-maximum intensity; the mean ± SEM of N vessels is indicated by the horizontal bar for each curve. a: depicts adventitia side of vessel wall; l: depicts intra-luminal side of vessel wall. (**D**) Comparison of fluorescence intensity of MLC-p in the smooth muscle layer. Data are presented as Mean ± SEM. (**E**) Comparison of fluorescence intensity of SM-α-actin in the smooth muscle layer of control and serum treated SCA. Mean ± SEM. (**F**) Comparison of fluorescence intensity of phalloidin labeled actin filaments in the smooth muscle layer of control and serum treated SCA. Mean ± SEM. *p < 0.05, n = 6 for control and serum treated arteries respectively. a.u.: arbitrary unit.

The transmural distributions of MLC-p, SM-α-actin and phalloidin labeled actin filaments (Fig. 4A,B) were compared between the serum-treated and control arteries. As shown in Fig. 4C, a leftward shift was observed in the MLC-p profile in the serum-treated vessels, while the distribution of SM-α-actin and phalloidin labeling did not significantly change (Supplemental Fig. 3A,B). Note that in these plots, both the fluorescence intensity and the vessel wall thickness were normalized to 1. As immunocytochemical labeling is considered semi-quantitative and the fluorescent intensity can vary between experiments, normalizing the fluorescence intensity allowed us to compare fluorescence signal profiles of vessels from different experimental days. We also normalized the wall thickness in order to average the distribution profiles among different vessels. In addition to the shift in MLC-p distribution, a decrease in the fluorescence intensity of MLC-p and SM-α-actin was also observed in the serum treated (constricted) vessels (Fig. 4D,E). The fluorescence intensity of phalloidin labeling was found to be similar in the control and serum treated vessels (Fig. 4F). When comparing the profiles of MLC-p, SM-α-actin and phalloidin labeling, the MLC-p distribution is shifted to the right of that for SM-α-actin in control vessels (Supplemental Fig. 4A), however, this shift is not observed in the serum treated group. (Supplemental Fig. 4B). In both groups, the distribution of phalloidin labeling was found to differ significantly to that of MLC-p and SM-α-actin. (Supplemental Fig. 4) Collectively, these results suggest that serum-induced vasoconstriction is associated with a leftward shift of the MLC-p profile and a decrease in the MLC-p and SM-α-actin fluorescence intensities.

### Comparison of the ratio of MLC-p/total MLC in control and serum vessels

**Figure 5:**
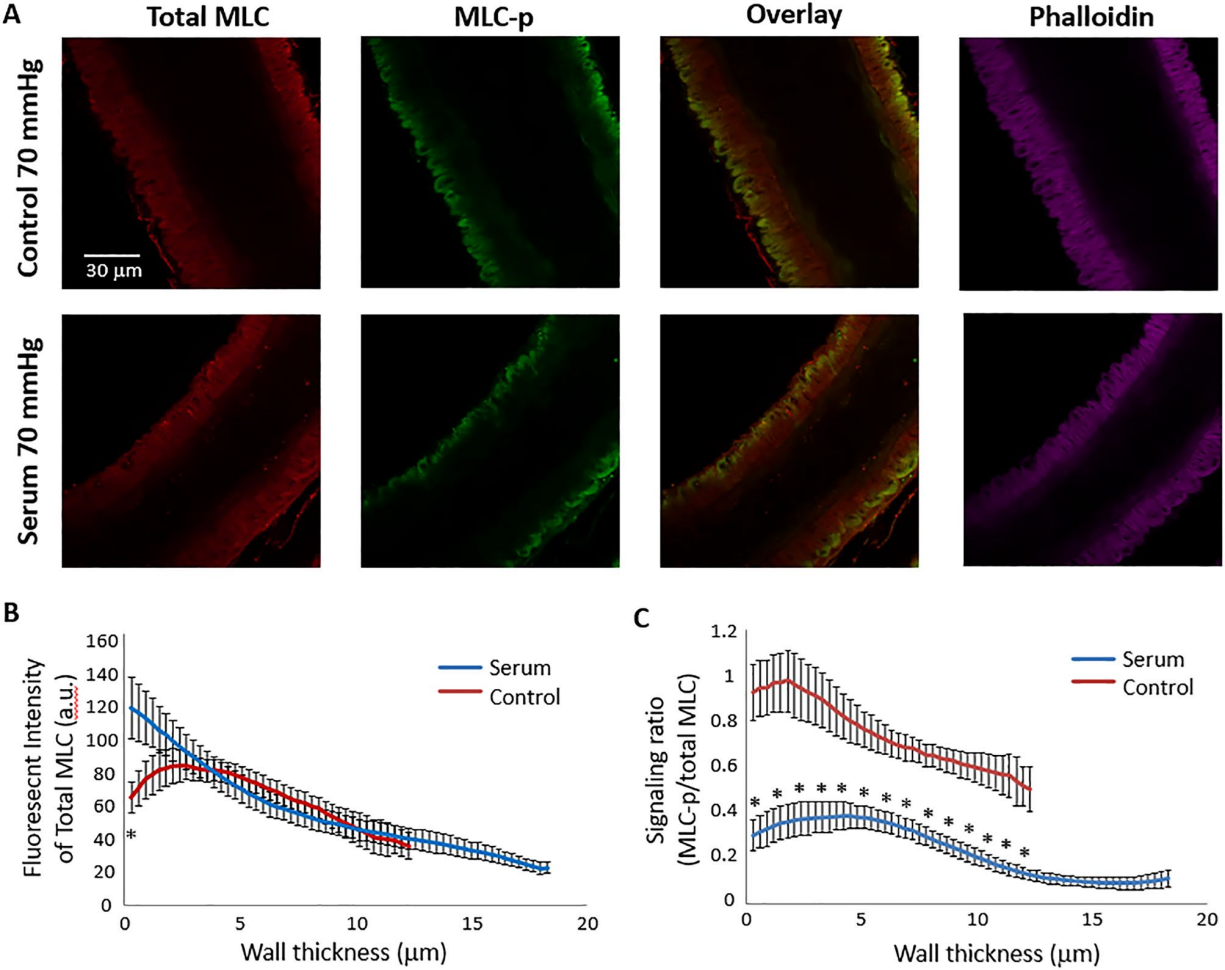
Comparison of total regulatory MLC and MLC-p labeling in control and serum constricted SCA. (**A**) Typical confocal fluorescence images of control and serum-treated rat SCAs immunolabeled for MLC-p, total regulatory MLC, and phalloidin-labeled actin filaments. (**B**) Comparison of total regulatory MLC fluorescent intensity in the vessel wall of control and serum treated rat SCAs. Mean ± SEM. (**C**) Comparison of MLC-p/total MLC ratio in the smooth muscle layer of control and serum treated SCAs. Mean ± SEM. *p < 0.05 between control and serum treated vessels. N = 6 vessels for control and serum treated groups respectively.

To further investigate the transmural distribution of phosphorylated MLC under control conditions and following serum-induced vasoconstriction, we simultaneously stained total regulatory MLC (total MLC) and phosphorylated MLC (MLC-p) in the vessel wall of both groups. As shown in Fig. 5A, total MLC labeling spanned across the thickness of the vessel wall, whereas the distribution of MLC-p labeling was limited to the outer layers of smooth muscle cells. In serum treated vessels, the total MLC labeling was greatest at the very outer layer of the vessel wall, exhibiting a different pattern from that observed in the control vessel (Fig. 5B). The fluorescence intensities of MLC-p and total MLC were used to calculate the ratio of MLC-p/total MLC for each vessel. It should be acknowledged that, due to the nature of immuno-fluorescence microscopy, this ratio cannot represent the actual level of MLC phosphorylation in the vessel wall, but allows us to observe variations in MLC phosphorylation level over the thickness of the vessel wall (i.e. transmural), and to compare the level of MLC phosphorylation between the control and serum treated groups. As shown in Fig. 5C, the MLC-p/total MLC ratio varies across the wall thickness and was significantly lower in the serum treated vessels compared with control SCAs. This finding is consistent with the results presented in Fig. 4D, suggesting that a lower level of MLC phosphorylation occurred during the maintained phase of serum induced SCA vasoconstriction.

### Reversal of serum-induced SCA vasoconstriction restores MLC-p and SM-α-actin distributions

**Figure 6:**
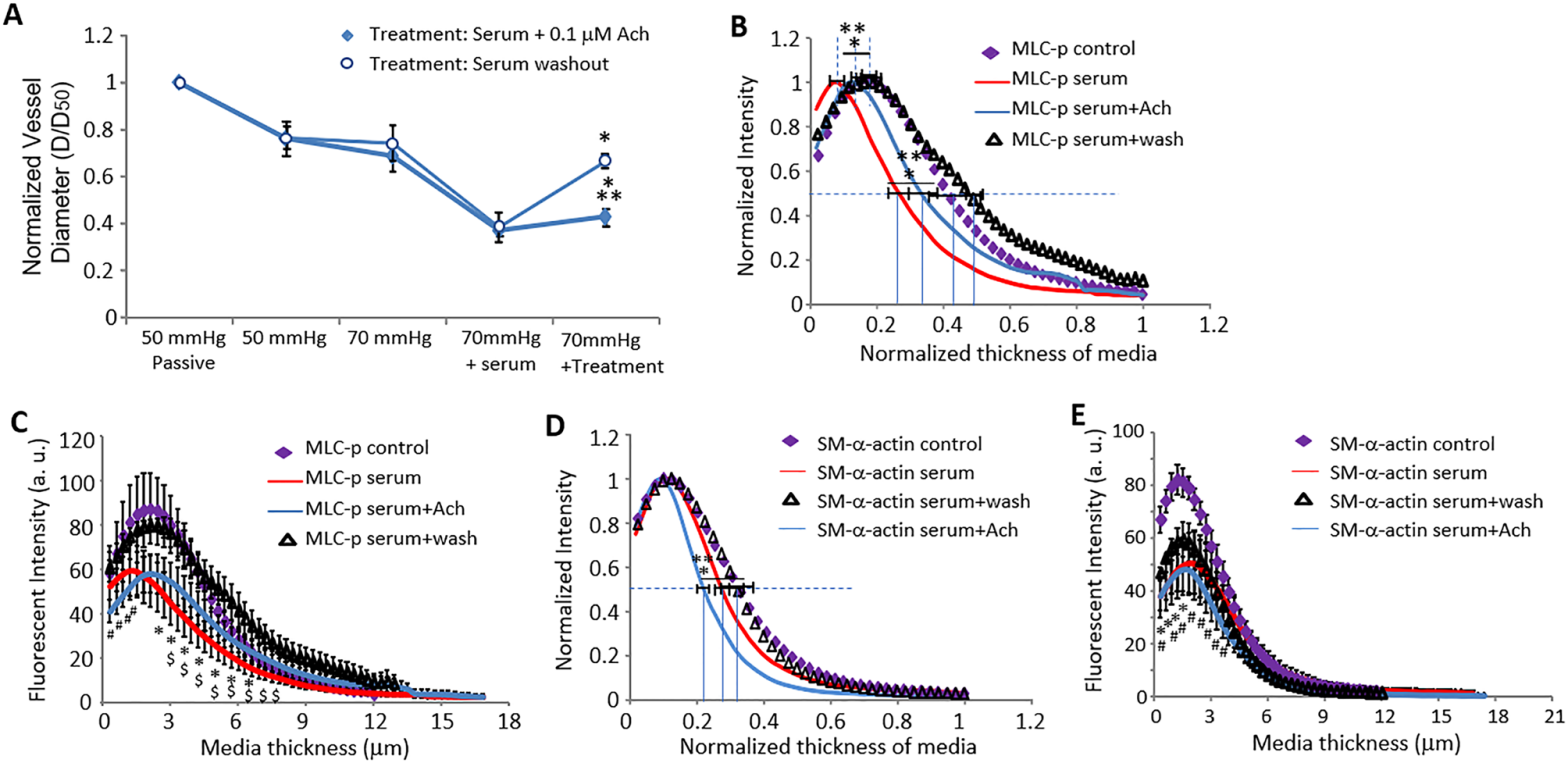
Reversal of serum induced SCA vasoconstriction. (**A**) Serum induced vasoconstriction of SCA was modestly inhibited by Ach (10^-7 M), but was largely reversed following washout of the added serum. D: vessel diameter 10 min after pressure elevation or after treatment; D_50_: passive vessel diameter at 50 mmHg. Data are presented as Mean ± SEM. *: *p* < 0.05 compared to vessel diameter at 70 mmHg + 10% serum, **p < 0.05 compared to 70 mmHg intraluminal pressure alone; n = 7 vessels for Ach treated group, and n = 5 for serum + wash group. (**B**) Normalized transmural distribution profile of MLC-p in contracted SCAs at 70 mmHg pressure under basal conditions, in the presence of 10% serum, serum + 0.1 μM ACh or following serum washout. The MLC-p profile shifted back to the distribution observed under control conditions after serum washout; while treatment with Ach induced no significant shift of MLC-p profile compared with treatment by serum alone. Data are presented as Mean ± SEM. *p < 0.05 compared to control; **p < 0.05 compared to serum washout. (**C**) The absolute intensity of MLC-p fluorescence across the arterial wall decreased in the presence of either 10% serum alone or serum + 0.1 μM ACh, and recovered to control levels following treatment washout. Data are displayed as Mean ± SEM. n = 6 vessels for control and serum-treated group respectively; n = 7 for serum + Ach and n = 4 for serum washout group. #*p* < 0.05, serum + Ach vs. control group; *p < 0.05, serum vs. control group; $*p* < 0.05, serum vs. serum washout group. (**D**) Normalized transmural distribution of SM-α-actin in SCAs at 70 mmHg pressure under basal conditions, and in the presence of 10% serum alone or serum + 0.1 μM ACh. Addition of Ach led to a further shift of the SM-α-actin profile to the left compared with serum constricted vessels. Serum washout reversed these observed shifts in SM-α-actin distribution to the control profile. *p < 0.05, serum + Ach vs. control; **p < 0.05 serum + Ach vs. serum washout. (**E**) The addition of either 10% serum alone or serum + 0.1 μM ACh decreased the intensity profile of SM-α-actin fluorescence across the arterial wall, which remained altered following treatment washout. Data are displayed as Mean ± SEM. *p < 0.05, serum vs. control group; #*p* < 0.05, serum + Ach vs. control group. n = 5 vessels for control, serum, serum washout and serum + Ach group respectively.

We further examined if the shifted MLC-p profile could be restored by reversing the serum-induced vasoconstriction. Treatment with the endothelium-dependent vasodilator acetylcholine (Ach, 0.1 μM) caused only a moderate (~ 13%) but statistically significant dilation of the serum-induced constriction, while wash-out of serum with PSS buffer restored vessel diameter to ~ 90% level of its original myogenic tone (Fig. 6A). In the presence of Ach, the MLC-p profile shifted moderately to the right (Fig. 6B), while the profile of the SM-α-actin showed a moderate shift to the left (Fig. 6D), suggesting an effect of ACh-mediated vasodilatory signaling on the organization of the actin cytoskeleton in VSMC. Interestingly, with the vessel myogenic tone restored by wash-out of serum, the profile of MLC-p reverted to that observed in control vessels, and the fluorescence intensity of MLC-p was also increased to levels comparable to that of the control condition (Fig. 6B,C). On the other hand, removal of the serum was not associated with a statistically significant increase in fluorescence intensity for either SM-α-actin or phalloidin (Fig. 6E, Supplemental Fig. 5). These data collectively suggest that the reversal of the SCA vasoconstriction is accompanied by significant changes in the distribution of MLC-p and SM-α-actin, supporting the involvement of dynamic cytoskeletal rearrangements that appear to align with different types of contractile stimuli.

### Confirmation of MLCK Involvement in serum-induced SCA vasoconstriction

**Figure 7:**
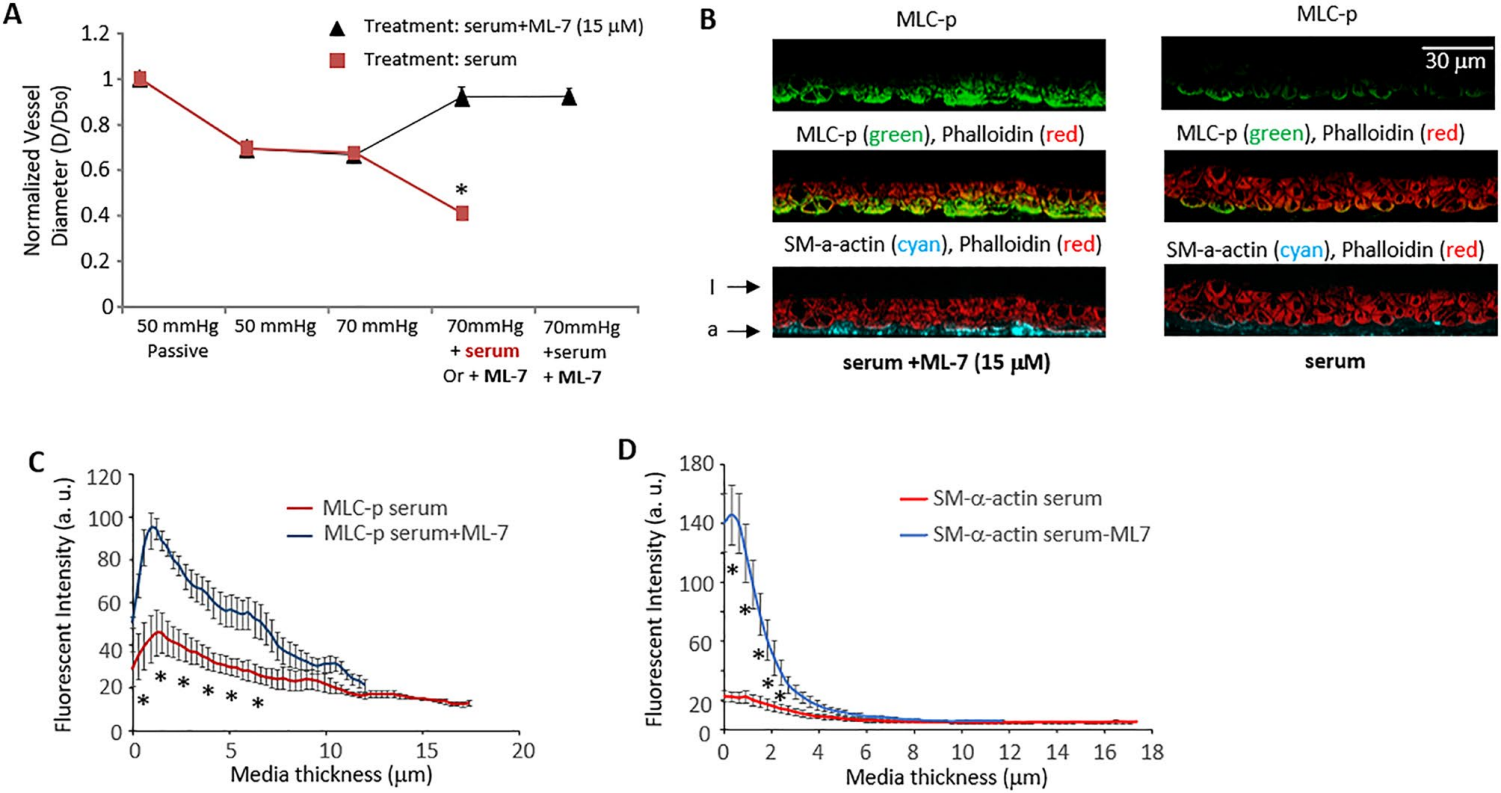
Pretreatment with ML-7 inhibited serum induced SCA vasoconstriction. (**A**) Serum induced vasoconstriction of SCA was abolished by ML-7 (15 μM) treatment. *D*_*50*_: passive vessel diameter at 50 mmHg, *D:* vessel diameter 10 min after pressure elevation or after treatment. *p < 0.05 compared to vessel diameter at 70 mmHg + 10% serum, n = 7 vessels for each group. (**B**) Comparison of MLC-p and SM-α-actin distribution in SCA dilated in the presence of ML-7 + serum vs. SCA constricted by serum. (**C**) The fluorescence intensity of MLC-p across the vessel wall of SCAs in the presence of ML-7 + serum vs. that of SCAs constricted by 10% serum. *p < 0.05 compared to serum treated SCA, n = 5 vessels for each group respectively. (**D**). The fluorescence intensity of SM-α-actin across the vessel wall of SCAs in the presence of ML-7 + serum vs. SCAs constricted with serum. Data are displayed as Mean ± SEM. *p < 0.05 compared to serum treated SCA, n = 5 vessels for each group respectively.

Blocking of MLCK activity using the kinase inhibitor, ML-7 (15 μM), abolished the spontaneous myogenic tone of rat SCA at 70 mmHg, and addition of 10% serum induced no further vasoconstriction (Fig. 7A). These observations are consistent with MLCK activity being required for serum induced vasoconstriction. However, an increase of MLC phosphorylation (Fig. 7B,C) and SM-α-actin (Fig. 7B,D) labeling is observed in vessels treated with serum plus ML-7 compared to the serum only treated group, suggesting that in the steady state maintenance phase, the levels of vasoconstriction may not be the major determinant of the levels of MLC phosphorylation and polymerization of SM-α-actin.

## Discussion

MLC phosphorylation and actin cytoskeleton reorganization are the main molecular mechanisms mediating the regulation of vascular tone in small arteries. However, much of our current understanding of these mechanisms relates to the acute phase of vessel constriction or dilation (i.e. the phase associated with active vessel diameter changes, and before a steady-state vascular tone is re-established), while less is understood about how MLC phosphorylation and the actin cytoskeleton are regulated when vessels maintain a level of stable tone. Intriguingly, our results suggest that a graded distribution of MLC phosphorylation and SM-α-actin cytoskeleton exists across the vessel wall of rat SCA, which peaks near the outer layer of VSMCs (i.e. towards the adventitial surface), and diminishes towards the inner layers of VSMCs (luminal surface). In the presence of rat serum (10%), ex vivo SCAs exhibited robust and sustained vasoconstriction, which could be reversed by wash-out of the serum, and inhibited by pretreatment with ML-7, while only modest reversal was observed in the presence of low does acetylcholine 0.1 (μM). Interestingly, after serum-induced vasoconstriction stabilized, we observed a shift of the MLC-p profile towards the outer edge of the vessel, as well as a decrease in the SM-α-actin and MLC-p fluorescence intensities. The changes in MLC-p intensity and transmural distribution were reversed by removal of the serum, while partial dilation with Ach was accompanied by a left shift of the SM-α-actin transmural distribution. Collectively, these fluorescence-labeling results reveal a spatial regulation of MLC phosphorylation and SM-α-actin in the vessel wall, both in the control condition and following serum-induced vasoconstriction.

The finding of a graded distribution of MLC phosphorylation and SM-α-actin across the vessel wall is novel and somewhat surprising. This pattern was not observed for the immuno-labeling of MHA, GFAP, desmin and pan-actin in the vessel wall, suggesting that antibody diffusion is not a limiting factor in the labeling of target proteins across the vessel wall. Prior analysis of MLC phosphorylation in cannulated resistance arteries has been limited by the small size of these vessels. Previously, a refined western blot technique had been developed by Takeya et. al. to evaluate the MLC phosphorylation in single arterioles^31^. Although highly sensitive, this technique does not allow detection of the spatial distribution of MLC phosphorylation within the vessel wall. The present study demonstrates that immuno-fluorescence microscopy is another sensitive and valuable tool for assessing MLC-p and SM-α-actin in single cannulated small arteries, with the added benefit of spatial resolution. The graded distribution of MLC phosphorylation has been previously reported across the left ventricular wall of the heart in a pattern similar to that observed in our SCAs, with a high MLC-p level at the outer wall that fades towards the inner wall^32^. In cultured gerbil fibroma cells, Peterson et.al. have shown a concentration of MLC-p at the cell periphery that was significantly greater than in the central region of the cell, while treatment with calyculin A (an inhibitor of myosin phosphatase) further enhanced the concentration of MLC phosphorylation at the cell periphery^33^. The graded distribution of MLC-p within the vessel wall perhaps challenges the current assumption that sustained vascular tone is achieved through uniform contraction of vascular smooth muscle cells and suggests there is a more refined control of smooth muscle contraction within the vessel wall.

It should be noted that the graded distribution of MLC-p in constricted SCAs is observed with vessels maintaining stable vascular tone. Whether there is spatial regulation of MLC phosphorylation during the acute phase of stimulated vasoconstriction is still unknown. Although calcium-mediated MLC phosphorylation has long been recognized as the central mechanism controlling VSMC contractile force^34^, how this process is regulated in small vessels maintaining a stable level of tone has not been thoroughly investigated. Earlier studies in larger SM tissues have established that an increase of MLC phosphorylation is necessary for the generation of smooth muscle force/stress, although this process does not follow a linear relationship, e.g. the fast contraction phase is usually associated with a higher level of MLC-phosphorylation, while the force-maintenance phase (“latch-state” or steady-state phase) is usually associated with a lower level of MLC-phosphorylation^34,35^. After carefully examining the levels of MLC-p and smooth muscle contractile force at steady-state, Ratz et. al. and Rembold et. al. have demonstrated that the smooth muscle contractile force (stress) is positively correlated with the level of MLC phosphorylation^36,37^. In myogenically active small arteries maintaining stable vascular tone, the mechanical output of VSMCs is usually measured by the degree of wall tension using LaPlace’s law. In earlier studies, we reported that MLC phosphorylation in small resistance arteries is positively correlated with wall tension^38^, which is consistent with the relationship found in the smooth muscle layer of larger arteries^36,37^. Using a refined western blotting technique^31^, Johnson et al.^39^ and Moreno-Dominguez et al.^40^ have also shown that the degree of MLC phosphorylation in cannulated cerebral and skeletal muscle resistance arteries was increased when intraluminal pressure was raised from 10 to 60 and 80 mmHg, which is likely accompanied by a significant increase of wall tension in these vessels. These studies, together with earlier observations, suggest that smooth muscle MLC phosphorylation is likely positively correlated with the vessel wall tension in small arteries.

This suggestion could, however, be counter-intuitive in the case of agonist/reagent-induced isobaric vasoconstriction. It is widely assumed that VSMCs will generate greater force during vasoconstriction, which is partly based on the many observations made using isometric tension myography with either arterial rings or vascular smooth muscle strips, where vasoconstrictor agonists induce an increase of contraction force^41–44^. However, it is often not fully considered that the mechanics of smooth muscle contraction recorded during isometric myography differ substantially from those of cannulated and pressurized vessels, i.e. there is no pressure stimulus and VSMCs are not allowed to shorten during isometric contraction. While the estimation of contractile force is directly measured by the force transducer in a wire myography instrument, in the cannulated and pressurized vessel, the contractile force can be estimated by wall tension^38,45^. In the maintained phase (or steady-state) of vasoconstriction for a cannulated artery, the vessel diameter stabilizes, indicating that an equilibrium has been reached between the contractile force generated by VSMCs and the wall tension imposed by transluminal pressure, thus allowing a direct estimate of VSMC generated forces using wall tension.

**Figure 8:**
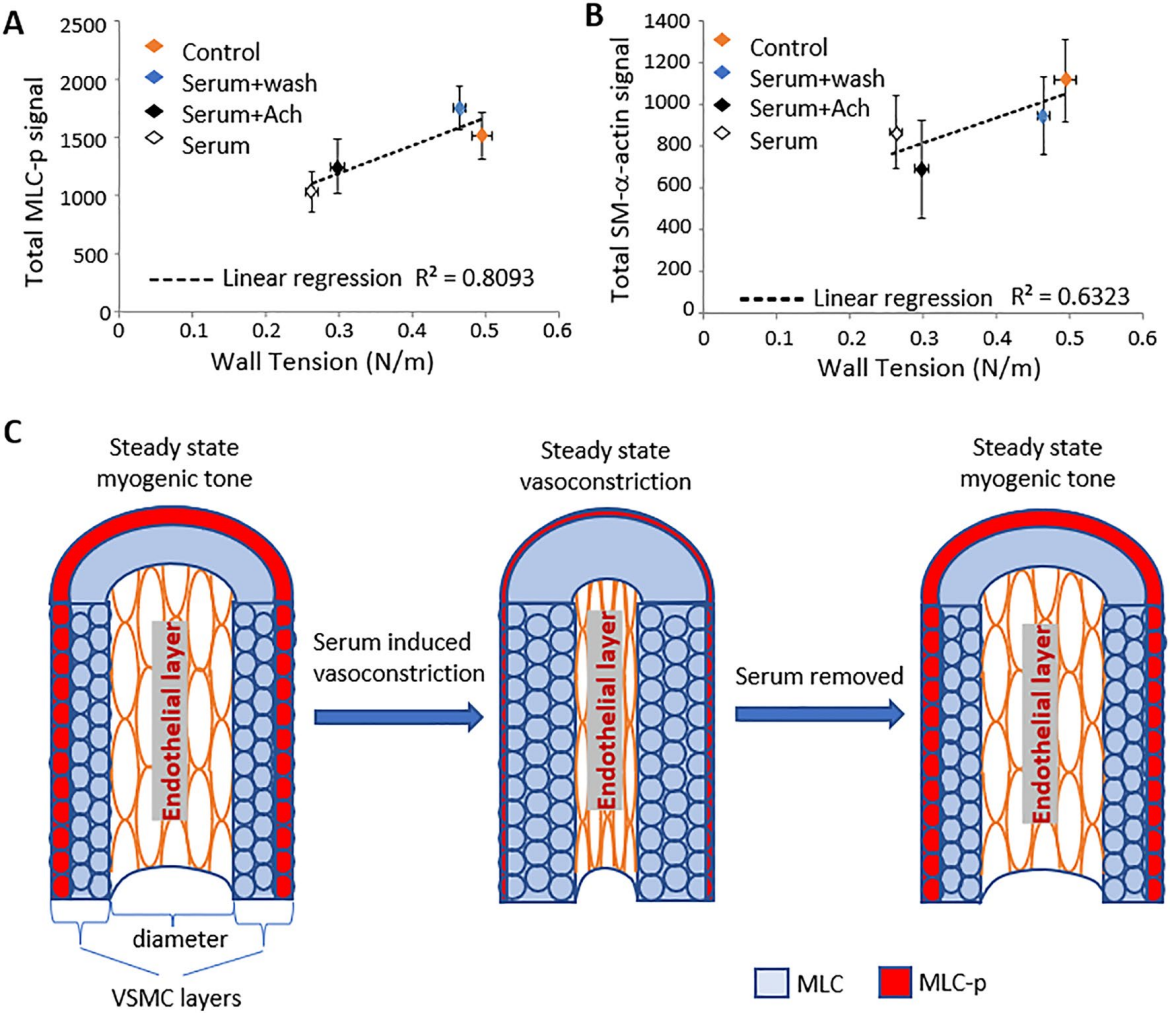
Correlation of wall tension vs. total MLC-p and total SM-α-actin among the four treatment groups of SCAs. Wall tension was calculated following LaPlace’s law, T = p*r, where T is wall tension, intraluminal pressure (p) is 70 mmHg, and r is the vessel radius measured before fixation. (**A**) Plot of wall tension vs. total MLC-p fluorescence intensity. (**B**) Plot of wall tension vs. total SM-α-actin fluorescence intensity. Data are presented as Mean ± SEM; Wall tension: n = 12 vessels each for control and serum-treated groups; n = 7 for the serum + Ach group and n = 5 for the serum washout group. Total MLC-p: n = 6 vessels each for the control and serum-treated groups; n = 7 for the serum + Ach group and n = 4 for the serum washout group. Total SM-α-actin: n = 5 vessels each for control, serum, serum washout and serum + Ach groups. (**C**) Schematic showing the observed changes of MLC phosphorylation during stable myogenic tone, serum induced vasoconstriction and re-established stable tone after serum washout.

According to LaPlace’s law, **T** = p*r, where “**T**” is wall tension, “**p**” is pressure and “**r**” is vessel radius. In the current study, sustained serum-induced vasoconstriction in SCAs led to a smaller “**r**” with no change in luminal pressure (70 mmHg). As such, the calculated wall tension (“T”) of the constricted vessel, would be expected to be smaller than that of the control vessels. Thus, the VSMCs in the control vessel need to generate greater force to oppose the resulting wall tension, compared to the serum-constricted vessel, contrary to what might be intuitively assumed. It should be noted that this conclusion only applies to the maintenance phase of vasoconstriction, and does not necessarily describe the active vasoconstricting process. Previous studies of vasoconstriction mostly focused on the active constricting phase, where agonist-induced vasoconstriction is accompanied by either an increase in cytosolic free calcium in VSMCs^46,47^ or a decrease of myosin light chain phosphatase (MLCP) activity^48,49^, both of which will presumably promote MLC phosphorylation and hence force generation. The discrepancy between these results and the current observation suggested that the regulation of MLC phosphorylation in VSMC could be different during the active constricting phase vs. the maintenance of vasoconstriction, which would suit the changing mechanical demands of each condition. There is currently no clear answer to how this transition is achieved at the molecular level, suggesting this is an overlooked component of our understanding of vasoconstriction. MLC phosphorylation and cytoskeletal organization are key mechanisms linking agonist-induced signaling and VSMC force generation, hence investigating these processes in the wall of cannulated resistance arteries will facilitate answering the questions raised above. Our results indicate that in cannulated rat SCA, and in the conditions described here, the control vessels had a higher level of MLC phosphorylation compared to serum-treated SCAs, and that the MLC phosphorylation is positively correlated with wall tension among the four groups tested (Fig. 8A,B), which is consistent with the previous findings in smooth muscle of conduit arteries^36,37^.

Aside from the acto-myosin crossbridge cycling, there is increasing evidence suggesting that dynamic actin cytoskeleton remodeling also plays an important role in the regulation of smooth muscle tension and vessel tone. Specifically, RhoA-, Rho-associated kinase- and PKC-mediated signaling pathways have been identified to regulate the actin polymerization/reorganization in vascular and airway smooth muscles^40,50–53^. In the current study, the different distribution of SM-α-actin and phalloidin labeled actin fibers is consistent with spatial regulation of actin isotypes in the vessel wall. Although relatively little is known regarding this type of regulation in VSMC^15^, in cultured fibroblasts, it has been shown that the incorporation of SM-α-actin into stress fiber depends on an AcEEED sequence at the N-terminal of SM-α-actin^54^. Interestingly, it has also been shown that incorporation of SM-α-actin into stress fibers depends on the substrate stiffness and the formation of super-mature focal adhesions, i.e. the incorporation of SM-α-actin could be inhibited when cells were cultured on substrates with a compliance < 11 kPa^55^. In our experiments, the wall tension of the serum treated vessels was ~ 0.26 N/m, corresponding to an average wall stress of ~ 14 kPa; while in the control vessels, the wall tension level was around 0.49 N/m, corresponding to an average wall stress of ~ 40 kPa. Interestingly, the SM-α-actin signal in the vessel wall showed a weak positive correlation with the wall tension (Fig. 8B), consistent with the findings reported by Goffin et. al.^55^.

Serum induced SCA vasoconstriction was blocked by the MLCK inhibitor, ML-7, suggesting that MLCK activity is required for this process. However, the MLC phosphorylation is increased in the group treated with serum plus ML-7 compared to the serum only group, suggesting that the regulation of MLC phosphorylation in steady state dilated rat SCA could be more complex than previously assumed. It is conceivable that, despite ML-7 inhibition of MLCK activity, other MLC phosphorylation regulatory mechanisms, independent of MLCK, are activated in the vessel wall. Candidate signaling mechanisms may include ILK (integrin linked kinase)^56,57^, ZIPK (zipper interacting protein kinase) and Rho-ROCK^58^, which also may contribute to phosphorylation of MLC in the outer layer VSMCs. This result also appears to support the current finding that MLC-phosphorylation positively correlates with vessel wall tension in rat SCA that exhibits a stable level of tone. It is worth noting that how MLC phosphorylation is regulated in other types of small arteries still needs to be elucidated and it is possible that heterogeneity may exist.

The signaling mechanisms underlying the graded transmural distribution of MLC-p and SM-α-actin is currently unknown. However, the positive correlation between these entities and vessel wall tension (Fig. 8A,B) led us back to the early studies of Paul Johnson, who hypothesized that smooth muscle cells may sense changes in wall tension^59^. Figure 8C schematically illustrates the major finding of the current study that a lower level of vascular tone in pressurized rat SCAs, as indicated by intraluminal diameter (control and serum washout conditions), is associated with a higher level of MLC phosphorylation, while maintenance of an augmented vasoconstricted state (i.e., serum treated SCAs with a decreased intraluminal radius) is associated with a lower level of MLC phosphorylation. With the consideration that the outer layer of VSMCs in the arterial wall is subjected to more mechanical stretching than the inner layers^60^, we suggest that enhanced mechano-transduction in the outer layer of VSMCs requires enhanced localization of MLC-p and SM-α-actin. For example, the enhanced activation of mechanosensitive ion channels^61^ may lead to a higher cytosolic calcium level in the outer layer of VSMCs, which could promote higher levels of MLC phosphorylation. In addition, the activation of integrin-, cadherin- and Angiotensin II type I receptor-mediated mechanotransduction^13,62,63^ could also lead to localized contractile events that are likely to be associated with the activation of MLC phosphorylation. In this regard, the presence of VSMC adherens junctions in the wall of pressurized rat SCA was demonstrated by immunolabeling of β-catenin (Supplemental Fig. 6). Our understanding that the outer layer of VSMC bears more mechanical stretch would also suggest that there would be more incorporation of SM-α-actin into the stress fibers, while in the inner layer of cells, the mechanical stress is comparatively smaller, thus limiting SM-α-actin incorporation. On the other hand, endothelial-derived vasodilation factors (including NO, prostacyclins and EDHF(s)) may also contribute to, or modulate, the formation of the gradients of MLC-p and SM-α-actin within the vessel wall.

In the context of translationally-relevant, SAH, acute spasm of small cerebral vessels has been observed in vivo in rodent SAH models^26–28^. Our data are consistent with these observations, and suggest that small cerebral arteries that are exposed to serum diffusion may experience strong vasoconstriction during SAH, even if the blood vessels are not in the vicinity of the bleeding site, i.e. a much broader brain area could be under the risk of ischemic attack during an episode of SAH. Indeed, cortical infarction and apoptosis in areas that are remote from the bled artery have been described in autopsy studies of SAH subjects^22,23^. In this aspect, the results described here could potentially be a contributing mechanism for the early arterial spasm and cortical infarction observed in SAH animal models. Our results further suggest that there is substantial cytoskeletal reorganization involved in the serum-induced vasoconstriction of SCAs.

One of the limitations of this study is that it remains unclear as to how serum triggers vasoconstriction of rat SCA. Deng et.al. have reported that serum induces MLC phosphorylation of human VSMC via the activities of ZIPK and ROCK, but independently of MLCK activity^58^. A number of vasoactive factors are known to be present in serum, such as PDGF, endothelin-1, Angiotensin II, serotonin, and Sphingosine-1-phosphate etc., and their concentrations could vary in wide ranges^64^. To resolve whether a combined effect of these factors or an unknown mechanism induced the sustained vasoconstriction is currently beyond the scope of this study. The fact that both the rat serum and bovine serum could induce vasoconstriction suggests that this could be a high probability phenomenon and worthy of further investigation. Another limitation is that this study relied heavily on one experimental technique, i.e. the use of immunofluorescence microscopy. Practically, other techniques are not readily available to resolve the spatial distribution of MLC phosphorylation and SM-α-actin in the vessel wall of small arteries. Finally, as the current experiments were performed on vessels from young male rats, it will be important to determine whether the results are applicable to females and aged animals.

In conclusion, the results presented here demonstrate that there is a previously unknown gradient distribution of phosphorylated MLC and SM-α-actin across the vessel wall of rat SCAs under steady state myogenic tone. These novel findings add to the suggestion that components of the signaling pathways controlling VSM contraction and cytoskeletal organization are regulated in a spatially defined pattern. Furthermore, MLC phosphorylation appears to correlate with vessel wall tension of rat SCA rather than the level of vasoconstriction, suggesting that the regulation of MLC phosphorylation could be different during the sustained phase of contractile tone compared to the process of active vessel constriction.

## Methods

All animal euthanasia and surgery procedures were conducted in accordance with protocols (Protocol No. 6656 and 8370) approved by the Institutional Animal Care and Use Committee of the University of Missouri, USA and conformed to the Guide For the Care And Use Of Laboratory Animals published by the US National Institutes of Health (NIH publication No. 85–23, 2011). The reporting in the manuscript follows the recommendations in the ARRIVE guidelines.

### Vessel isolation, myogenic response and vascular diameter measurement

Male Sprague–Dawley rats (200 ~ 250 g of weight) were used for all experiments. Following anesthesia by intraperitoneal injection of sodium pentobarbital (10 mg/100 g body weight) and euthanasia by pneumothorax, the brain was removed and placed in cooled (4 °C) dissection buffer. The superior cerebellar artery (SCA) was then isolated and cannulated between two glass micropipettes in a vessel chamber (Living System, Inc., Burlington, VT, USA) containing physiological saline solution (PSS, in mM: 145 NaCl, 5.6 KCl, 2 CaCl_2_, 1 MgSO_4_, 1.2 NaH_2_PO_4_, 3 MOPS, 5 glucose and 1 pyruvic acid). For each animal, one SCA vessel was used. A chamber heater and a pressure control unit (Living system, Inc.) were used to control the vessel temperature (37 °C) and lumen pressure, respectively. Bath solution was changed every 10 min during an experiment. Vessel intraluminal pressure was initially set to 50 mmHg (in the absence of flow). and passive vessel diameter at 50 mmHg was measured in the absence of Ca^2+^. After establishing spontaneous myogenic (intrinsic) tone, pressure was raised step-wise to 70 mmHg (and then 90 mmHg in limited experiments). Each pressure step was maintained for 10 min to ensure that the vessel had achieved a stable lumen diameter. For serum exposure experiments, after vessel diameter stabilized at 70 mmHg, the bath solution was replaced with PSS containing 10% Rat serum (Atlantic Biologicals, Inc.) or PSS alone (negative control). For ML-7 experiments, after vessel spontaneous tone stabilized at 70 mmHg, the bath solution was replaced with PSS containing 15 μM ML-7. After 10 min of treatment, the bath solution was replaced again with PSS containing 10% rat serum and 15 μM ML-7. Images of the vessel segments were recorded using a video camera and vessel lumen diameter measured offline using an edge-detecting software written in Matlab.

### Vessel immunofluorescence labeling

For immuno-labelling, SCA were fixed with 8% paraformaldehyde (PFA) at steady-state for 1 h with transmural pressures of 70 mmHg. A relatively high concentration of PFA was used with the goal of achieving rapid fixation across the cell layers in the vessel wall. Vessels were then incubated in glycine buffer (0.1 M glycine in MilliQ water) for 1 h (2×) to quench remaining paraformaldehyde. Vessels were subsequently exposed to a primary antibody buffer (1% BSA (w/v), 0.05% Triton X-100 (v/v) in sodium citrate solution buffer (NaCl:152 mM, sodium citrate: 17 mM, pH 7.4)) plus primary antibody with agitation at 37 °C for 48 h. The vessels were then washed 3 × with buffer (0.05% Triton X-100 in sodium citrate solution buffer) and incubated in secondary antibody solution overnight (37 °C with agitation). After incubation with the secondary antibody, the vessel was washed 3× (0.05% Triton X-100 in sodium citrate solution), mounted on a coverslip, and imaged using confocal microscopy. Details of the antibodies used are provided in Supplemental Table 1. Confocal imaging of the vessel was performed on a confocal microscope (FV1000, Olympus, Inc., Long Island, NY) using a 60× oil objective with 1.42 NA. Through focus images were collected at 0.3 µm intervals. Vessels that were labeled with isotype control antibodies showed negligible fluorescence signal^13,62,63^.

### Quantification of fluorescence intensity across vessel wall

Vessel wall image stacks (with z-slice = 0.3 μm) were analyzed using Image J (NIH, Bethesda, MA). An orthogonal cross section was selected at the center bottom of the vessel (as shown in Fig. 1A). The average fluorescence intensity across the selected section was then calculated and plotted against the thickness of the vessel wall to determine the spatial profile of fluorescently labeled MLC phosphorylation, SM-α–actin and total MLC. Since immunofluorescence staining is considered semi-quantitative, several steps were taken to minimize any differences that could be caused by variation in vessel staining and image acquisition. For vessel staining, blood vessels from different treatment groups were batched processed, and labeled side by side with the same primary and secondary antibody buffer for the same length of time and were washed for the same time periods. The laser intensity and the gain settings of the imaging system were kept the same for all vessels processed in the same batch and were carefully controlled to avoid signal saturation. For comparisons of fluorescence intensity, only vessels labeled in the same batches were included.

### Antibody validation by western blot

Lysates of cultured VSMCs isolated from rat arterioles were separated by electrophoresis using 4–15% Criterion Precast Gels (Bio-Rad). For Western blot, the samples were probed using primary antibodies against phosphorylated MLC and SM-α-actin, followed by HRP-conjugated goat anti-rabbit or anti-mouse antibodies. See Supplemental Table 1 for antibody dilutions. The probed membranes were visualized using a ChemiDoc imager (Bio-Rad).

### Statistical analysis

Results are presented as Mean ± SEM. Statistical comparisons were made using ANOVA and Student ‘s T-test. Statistical significance was assumed at a *P* value less than or equal to 0.05.

## Supplementary Information

Supplementary Information.

## Data Availability

The datasets generated during and/or analyzed during the current study are available from the corresponding author on reasonable request.
