# Supplementary material for: Myosin light chain phosphorylation exhibits a gradient across the wall of cerebellar arteries under sustained ex vivo vascular tone
Source: Sci Rep. 2023 Jan 17;13:909. doi: 10.1038/s41598-023-28092-3 (PMC9845333; doi:10.1038/s41598-023-28092-3)
Supplement: Supplementary file 1 — Supplementary Information. [file 41598_2023_28092_MOESM1_ESM.pdf]

Myosin light chain phosphorylation exhibits a gradient across the wall of cerebellar arteries under sustained ex vivo vascular tone.

Zhe Sun[1,2], Zhaohui Li[3], Mackenna Rodgers[1], Liping Zhang[1] and Michael A Hill[1,2]

[1]Dalton Cardiovascular Research Center, [2]Department of Medical Pharmacology and Physiology, [3]Department of Radiology, School of Medicine, University of Missouri, Columbia, MO, U.S.A.

Brief Title: Heterogeneity of MLC phosphorylation across the artery wall

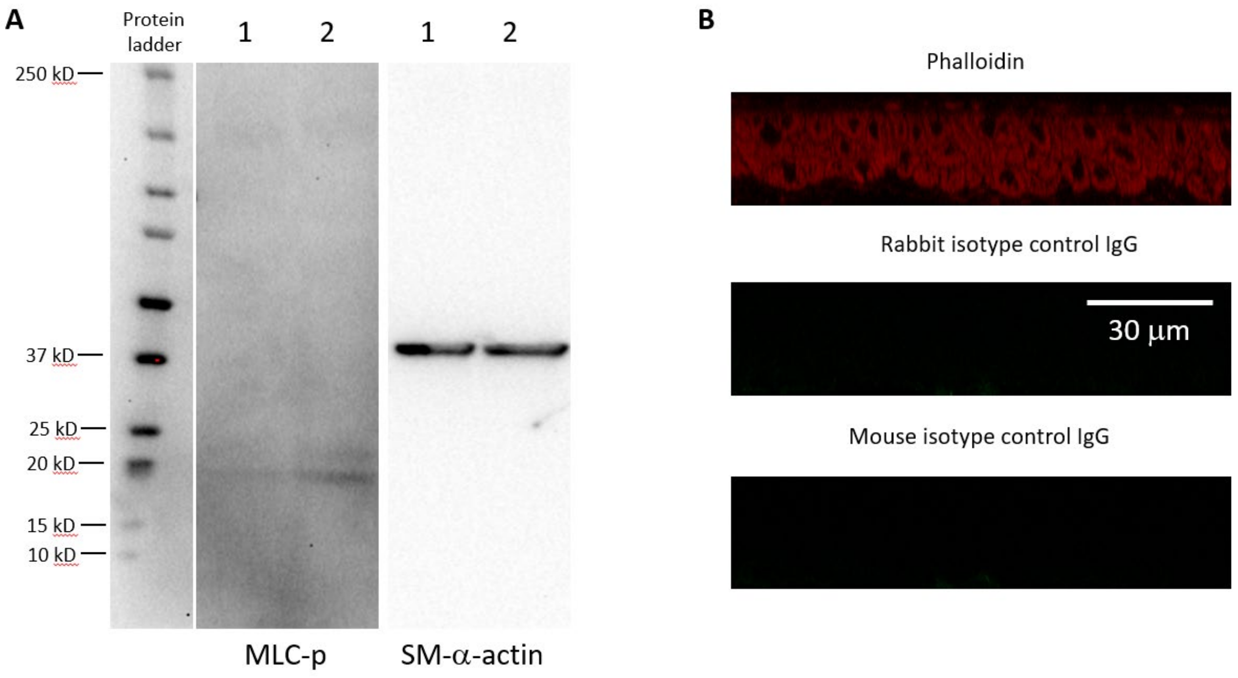

Supplemental Figure 1. Validation of the specificity of MLC-p and SM-α-actin immuno-labeling. **A.** Western blot for rat MLC-p and SM-α-actin using the selected antibodies. Cultured rat VSMCs were treated with either DMSO (Vehicle control, Lane 1) or U-46619 (10 nM, Lane 2) for 15 mins. The cell lysates were rapidly prepared after treatment and were subjected to SDS-PAGE. The membranes were first probed using the antibody against MLC-p, followed by stripping and re-probing using the antibody against SM-α-actin. The experiment was repeated 3

times, and typical results are shown. **B.** Immunofluorescent labeling of a control rat SCA pressurized to 70 mmHg using rabbit and mouse IgG isotype control antibodies with appropriate dilutions.

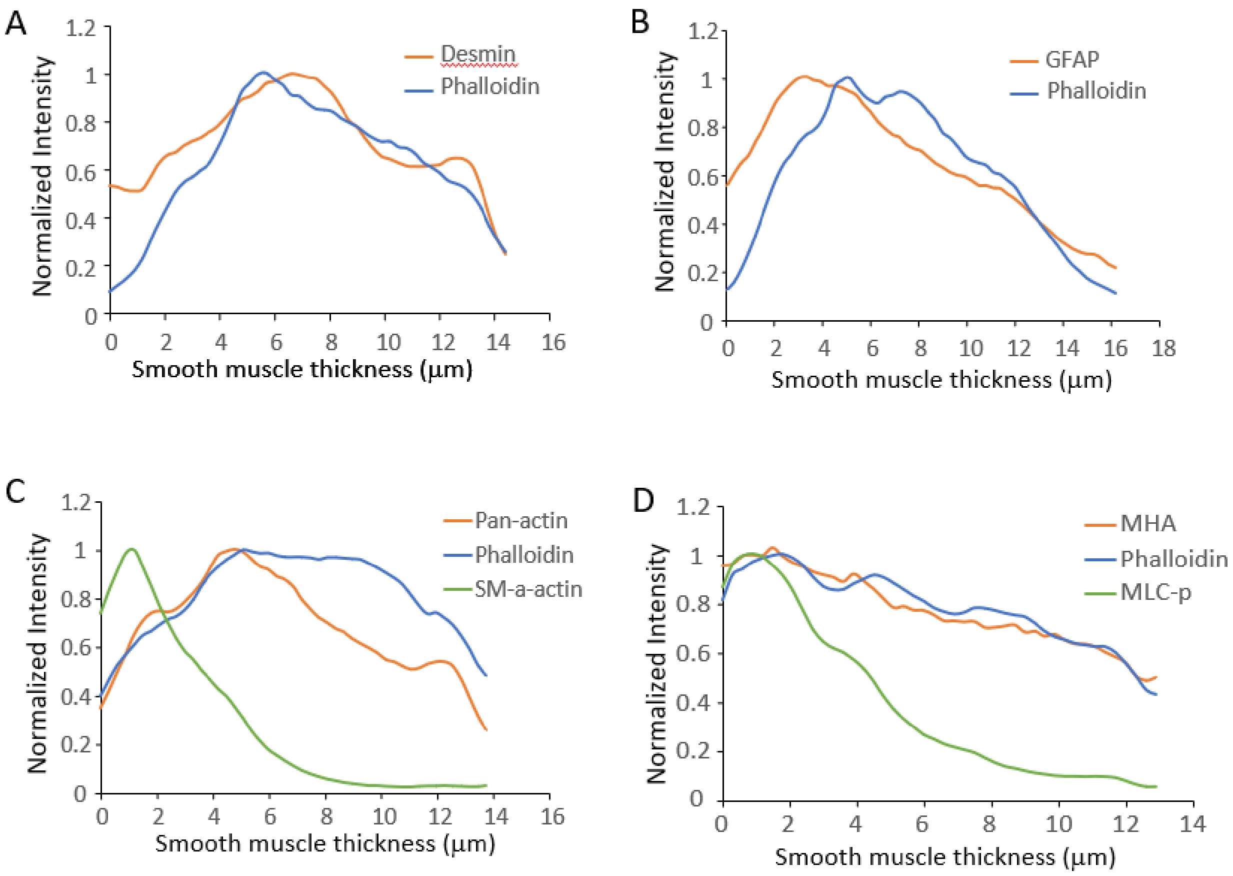

Supplemental Figure 2. Transmural intensity profile of fluorescence signals for various cytoskeletal proteins in the SCA vessel wall. (A) Desmin and (B) GFAP, (C) Pan-actin vs. SM-α-actin and (D) MLC-p vs. MHA.

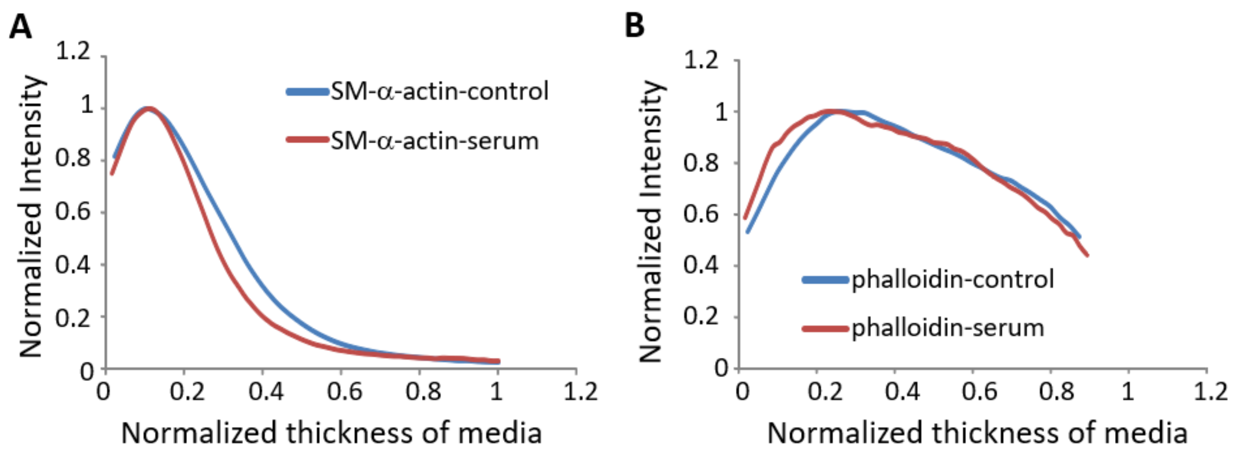

Supplemental Figure 3. Quantification of the transmural profiles of SM-α-actin and phalloidin in control and serum-constricted SCAs at 70 mmHg intraluminal pressure. **A.** Normalized distribution of SM-α-actin across the arterial wall in control and serum-treated groups. **B.** Comparison of the normalized phalloidin labeled actin filament profile in control and serum-constricted SCAs. n=6 for control and serum treated arteries respectively.

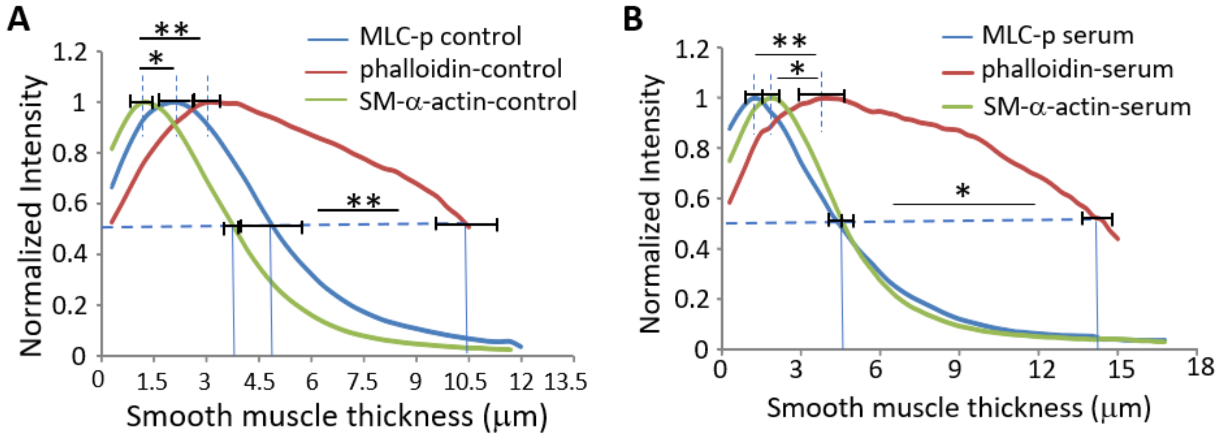

Supplemental Figure 4. Comparison of the transmural profiles of pMLC, SM-α-actin filament and phalloidin-labeled actin filaments in control (**A**) and serum constricted SCAs (**B**). Distribution profiles for the three markers were significantly shifted from each other in the smooth muscle layer of control SCAs. The dashed line indicates the points of half-maximum intensity for the three profiles. n=6 vessels for each group. Data are presented as Mean±SEM. *: $p<0.05$ compared to the MLC-p. **: $p<0.05$ compared to phalloidin.

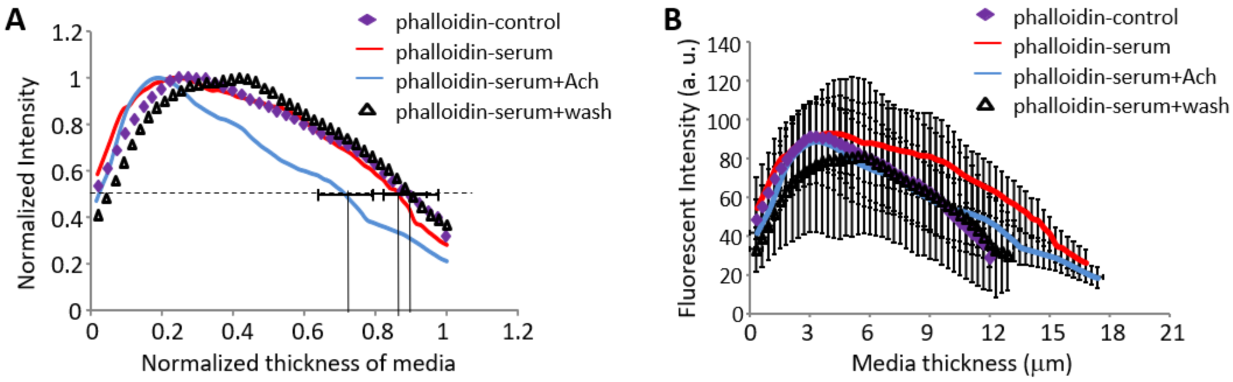

Supplemental figure 5. Effects of Ach addition and serum washout on the normalized transmural profile of phalloidin (A) and the fluorescence intensity of phalloidin labeling (B) in control and serum-treated SCAs. Data are displayed as Mean±SEM; n=5 vessels for control and serum-treated groups; n=7 for the serum+Ach group, and n=4 for the serum washout group. Treatment conditions did not induce statistically significant changes in either the transmural profile of phalloidin labeling (panel A) or the intensity of phalloidin fluorescence in SCAs.

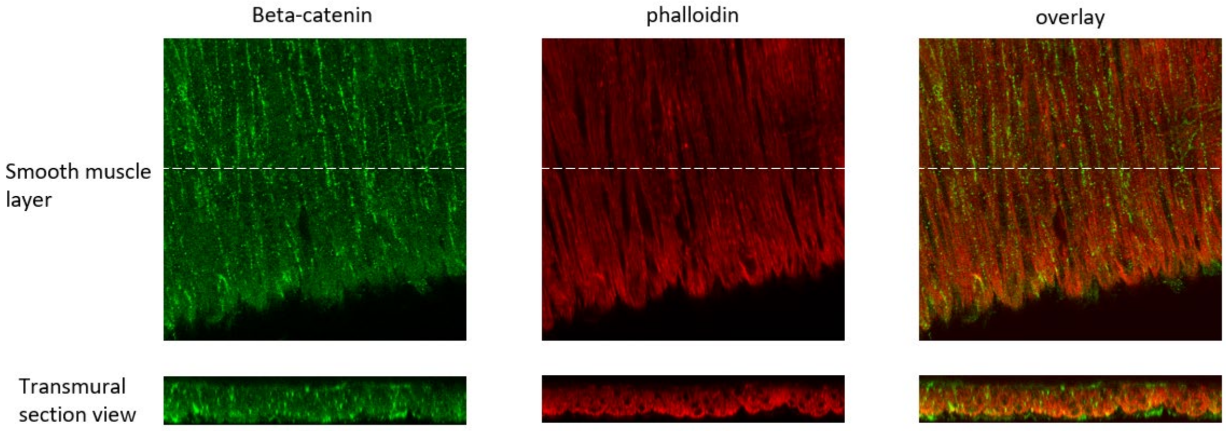

Supplemental Figure 6. Presence of adherens junctions in the SCA vessel wall. Vessels were pressurized at 70 mmHg and allowed to develop spontaneous tone. A. Immunofluorescence labeling of adherens junction protein beta-catenin (green), and actin stress fibers (red) in the smooth muscle layer of SCA. Transmural sectional view of the vessel wall at the position indicated by the white dashed line is shown at the bottom.

Supplemental Table 1. List of antibodies used in the vessel labeling

Antigen	Dilution ratio	Catalog number	Vendor
MLCp (Rabbit IgG)	1:100*	ab2480	Abcam
alpha-SMA (Mouse IgG)	1:100*	A5228	Sigma-Aldrich
total regulatory MLC (Mouse IgG)	1:100	MABT180	Sigma-Aldrich
Desmin (mouse IgG)	1:50	D1033	Sigma-Aldrich
GFAP (mouse IgG)	1:100	G3893	Sigma-Aldrich
phalloidin-Alexa 568	1:200	A12380	ThermoFisher
MHA (rabbit IgG)	1:100	MP3791	ECM biosciences
Pan actin (mouse IgG)	1:100	MAB 1501	Chemicon Internatonal
Alexa 488-Goat-anti-Rabbit IgG	1:200	A11008	Lifetechnology
Alexa 647-Goat-anti-Mouse IgG	1:200	A32728	ThermoFisher
Mouse IgG Control<br>1 mg/ml	1:100	I5381	Sigma-Aldrich
Rabbit IgG Isotype<br>2.5 mg/ml	1:250	3900S	Cell Signaling

*: 1:1000 dilution was used for western blotting experiments

VSMC VSMC

Lane Lane

1 2

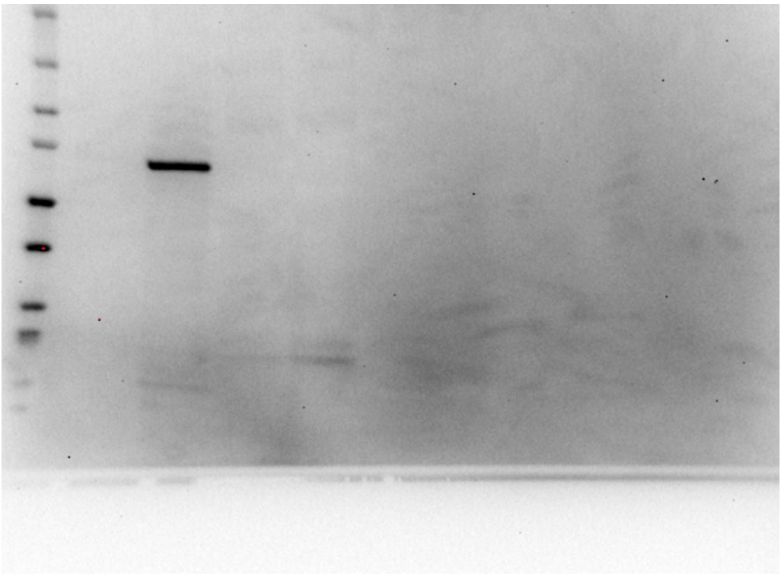

MLC-p western blot

whole membrane image

Colormetric

Protein

ladder

VSMC VSMC

Lane Lane

1 2

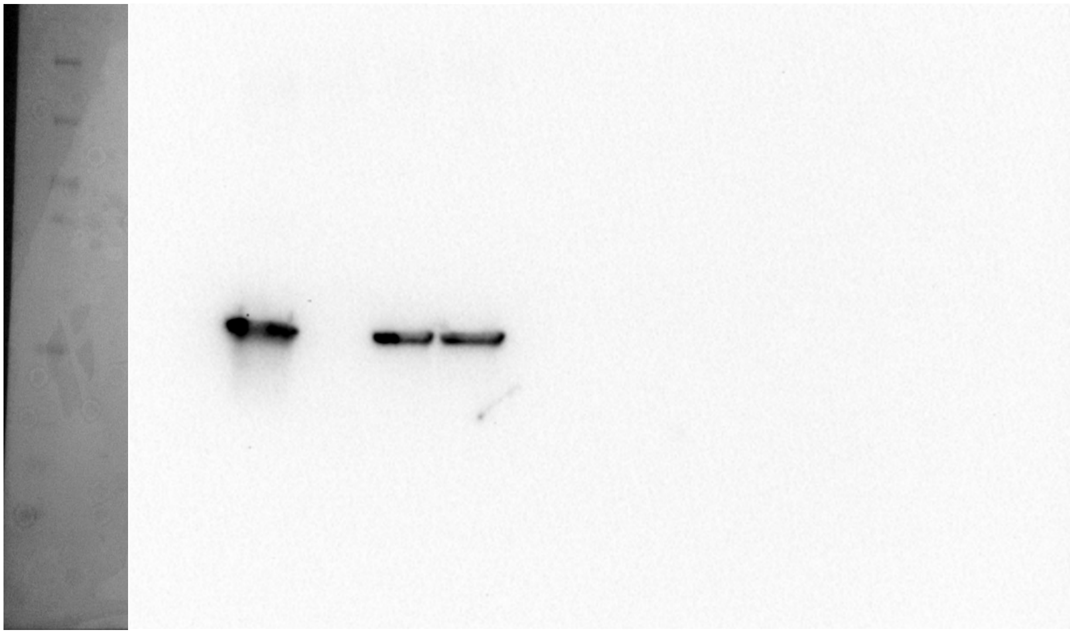

SM-α-actin

Western blot

whole membrane image

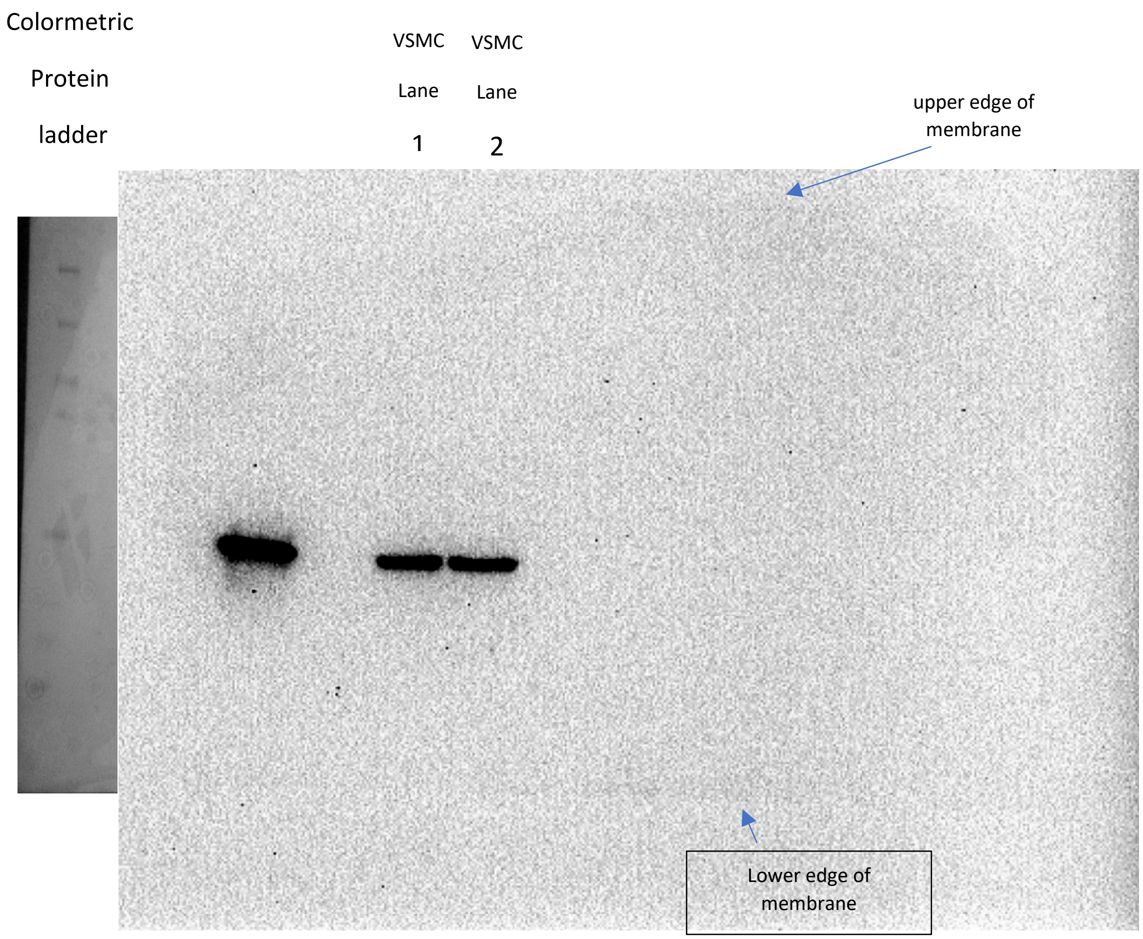

SM-α-actin

Western blot

whole membrane image
